# Migrated Inferior Vena Cava (IVC) Filter Presenting as Tricuspid Valve Mass, Right-Sided Heart Failure, and Parodoxical Emboli

**DOI:** 10.7759/cureus.41046

**Published:** 2023-06-27

**Authors:** Al Ameen Oredegbe, Matthew Derakhshesh, Hafiza Hareem Waqar, William Alderisio

**Affiliations:** 1 Internal Medicine, Albany Medical College, Albany, USA; 2 Internal Medicine, Albany Medical Center, Albany, USA; 3 Cardiovacular Medicine, Albany Medical Center, Albany, USA

**Keywords:** : ischemic stroke, atrial septal defect (asd) diagnosis, ivc filter migration, right-sided heart failure, tricuspid valve regurgitation

## Abstract

A 58-year-old male with an unknown medical history presented with acute encephalopathy, receptive aphasia, and hypertensive emergency. The patient did not have any family members from whom a collateral history could be obtained. He underwent X-rays of the abdomen and bilateral humeri/femurs to check for foreign bodies. He was found to have right femoral open reduction and internal fixation with retained screw fragments. He was diagnosed with ischemic stroke on MRI. Transthoracic echocardiogram (TTE) revealed right-sided heart failure and a tricuspid valve mass as well as right to left shunting. This raised concern for large atrial septal defect (ASD) with paradoxical embolization from tricuspid valve mass. Transesophageal echocardiogram (TEE) redemonstrated large ASD. Concern was raised for the ASD closure device as the cause of this "tricuspid mass." Due to history of orthopedic procedure, it was hypothesized that the patient had an IVC filter placed in the setting of pulmonary embolism (PE) prior to an orthopedic procedure. The tricuspid valve was visualized under fluoroscopy and was confirmed to be a migrated IVC filter. He was taken to the operating room (OR) for cardiac surgery for the removal of the IVC filter and repair of ASD. Surprisingly, no ASD was found.

## Introduction

Venous thromboembolism (VTE) is an important cause of morbidity and mortality among inpatients [1]. VTE is the leading cause of preventable hospital death in the United States. Anticoagulation is the mainstay of treatment and prevention for VTE. However, anticoagulation is contraindicated in patients with active bleeding, major trauma, intracranial hemorrhage, or recent high-risk surgery [2]. Inferior vena cava (IVC) filter placement is an option in these patients. The development of retrievable IVC filters has rapidly expanded their use. Unfortunately, these devices are associated with complications some of which are highly morbid [3]. The most common complications of IVC filter placement are device migration, fracture, and perforation. IVC filter migration refers to either migration of the entire device or of a fractured part to a distant location. One of the causes of filter migration is an IVC that is too large for the IVC filter; most filters are approved for IVCs that are 28 mm or less in diameter [4]. Migrated IVC filters can migrate and lodge in the intrahepatic IVC, superior vena cava, right heart, and pulmonary artery [5]. Migration to the right heart often results in tricuspid regurgitation, right heart failure, pulmonary infarction, and cardiac tamponade. In most of these cases, the patient has a known history of IVC filter placement and presents with symptoms and signs that raise suspicion for filter migration. We present the case of a patient with an unknown medical history presenting with signs of right heart failure in whom the presence of an IVC filter was deduced. The following case report details the diagnostic process that led to this discovery.

## Case presentation

A 58-year-old male presented with 1 day of confusion. He was found walking around in a neighbor’s backyard by the police and was brought to an outside hospital. He was not accompanied by family and he could not provide a history. In addition, available medical records were very limited.

Initial vital signs: temperature 36.3^o^C, blood pressure (BP) 182/104 mmHg, heart rate (HR) 87 bpm, respiratory rate (RR) 16 breaths/min, and oxygen saturation was 94% on room air.

Physical examination showed a paranoid and confused patient. He was unable to answer questions asked and his sentences did not make sense. He was disoriented to place, time, and situation. He had receptive aphasia but no dysarthria. He was unable to name common objects. Cardiac auscultation revealed a holosystolic murmur best appreciated in the left lower sternal border, augmented by inspiration. Lungs were clear to auscultation bilaterally with no crackles or wheeze. The abdomen was soft, non-tender, and nondistended. No hepatosplenomegaly. He had 2+ pitting edema in his bilateral lower extremities. The neurologic exam showed that strength was 5/5 in all extremities. Gait was grossly normal.

**Figure 1:**
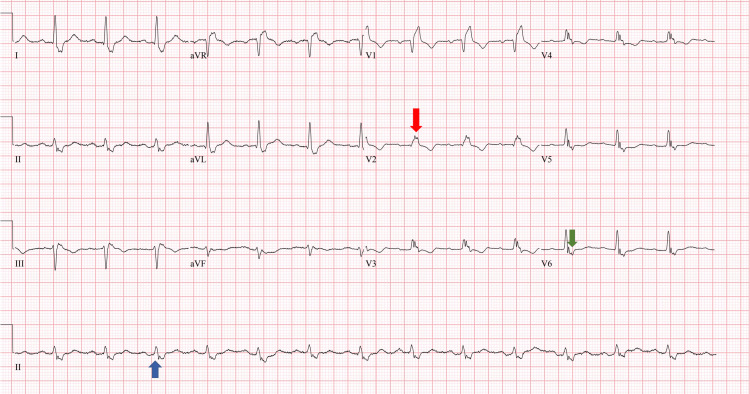
Initial EKG showing the RBBB. Figure 1 shows an EKG consistent with RBBB. The blue arrow highlights the prolonged QRS duration (>120 ms) in the rhythm strip typical of RBBB. The red arrow highlights an RSR' pattern of the QRS complex in the right-sided precordial leads (V1-V3). The green arrow depicts the wide-slurred S wave in V6 typical of RBBB. EKG, electrocardiogram; RBBB, right bundle branch block

Initial electrocardiogram (EKG) showed (see Figure 1): normal sinus rhythm. Right bundle branch block. Septal infarct of undetermined age.

Laboratory data on admission are given in Table 1.

**Table 1:** Laboratory data on admission. WBC, white blood cell count; RBC, red blood cell count; Na, sodium; K, potassium; Cl-, chloride; HCO3-, bicarbonate; BUN, Blood Urea Nitrogen; Cr, creatinine; eGFR, estimated glomerular filtration rate; AST, aspartate transaminase; ALT, alanine transaminase

Test	Result	Reference range
WBC	5.2	4.0-9.0 /uL
RBC	3.93	4.5-5.7 /uL
Hemoglobin	12.5	13.6-16.7 g/dL
Hematocrit	37.8	40.0-49.0%
Platelet count	92	130-350 /uL
Na	133	135-145 mEq/L
K	3.1	3.4-5.2 mEq/L
Cl-	99	99-109 mEq/L
HCO3-	27	21-30 mmol/L
BUN	12	7-22 mg/dL
Cr	0.86	0.8-1.4 mg/dL
eGFR	100	>60 mL/min/1.73 m^2^
Calcium	8.2	8.6-10.3 mg/dL
Alkaline phosphatase	90	30-115 IU/L
AST	23	5-45 IU/L
ALT	10	5-60 IU/L

**Figure 2:**
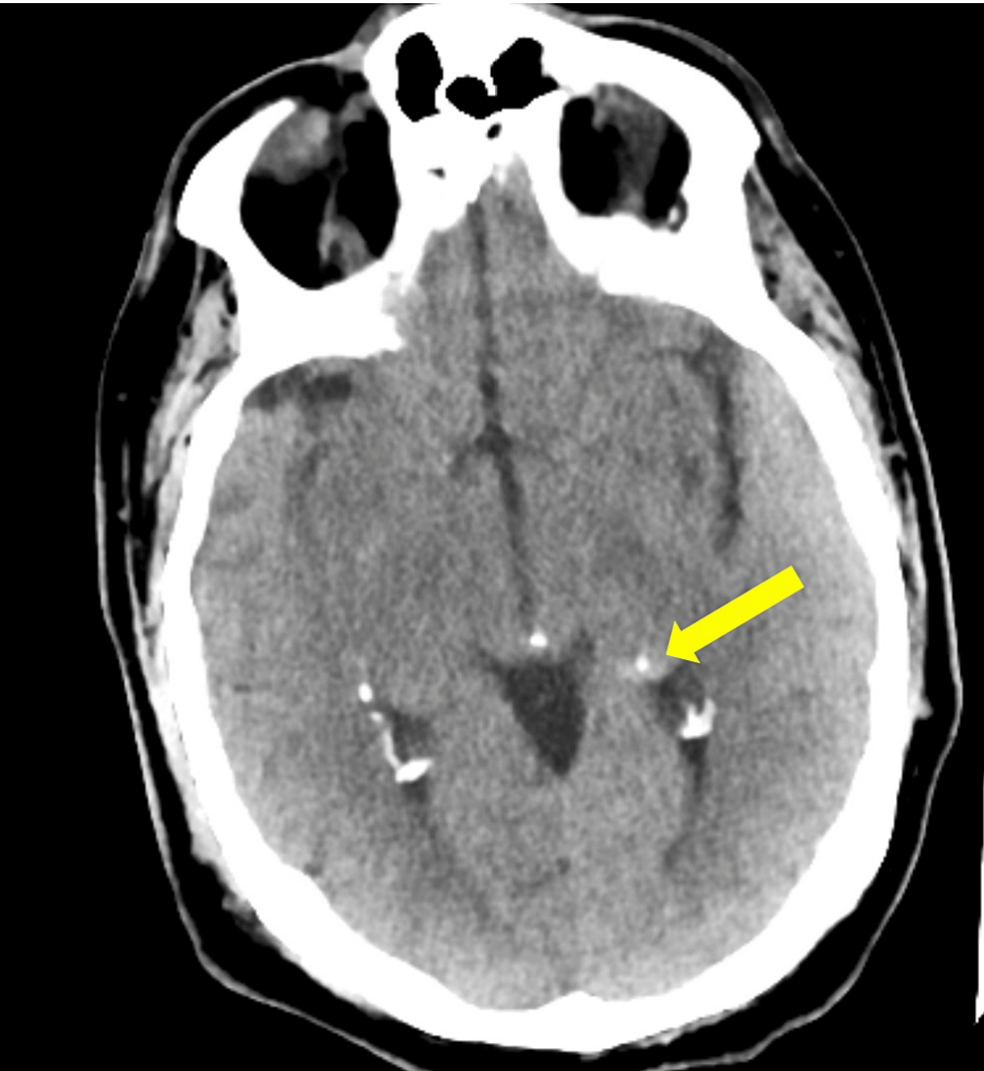
CT head showing hyperattenuation in the left hippocampus suggestive of calcification and/or mineralization. CT, computed tomography

A CT scan of the head showed hyperattenuation throughout the entire left hippocampus suggestive of calcification and/or mineralization. It did not show any signs of ischemic stroke. See Figure 2.

**Figure 3:**
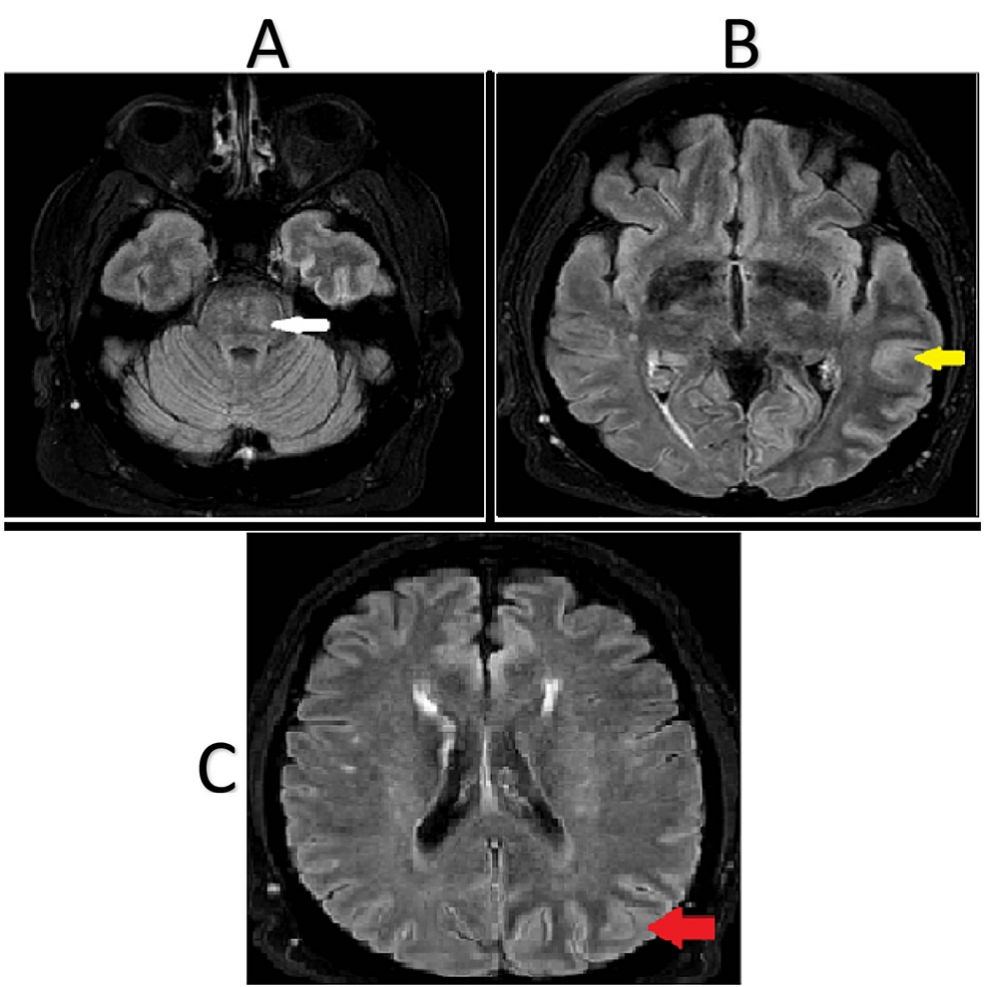
MRI brain showing increased FLAIR signal in the central pons, occipital, and temporal lobes. In image A, the white arrow highlights the increased FLAIR signal in the central pons. In image B, the yellow arrow depicts gyriform swelling and increased FLAIR signal in the left temporal lobe. In image C, the red arrow highlights gyriform swelling and increased FLAIR signal in the occipital lobe. MRI, magnetic resonance imaging; FLAIR, fluid-attenuated inversion recovery.

Three days into his admission, the patient had five witnessed tonic-clonic seizures lasting about 20 s. MRI brain showed focal restricted diffusion and increased FLAIR (fluid-attenuated inversion recovery) signal in the central pons raising concern for an acute or subacute pontine stroke. See Figure 3 below.

The MRI findings raised concern for herpes simplex virus (HSV) or limbic encephalitis. The patient was started on acyclovir. A lumbar puncture was performed and cerebrospinal fluid (CSF) analysis was inconsistent with encephalitis (see Table 2).

**Table 2:** CSF analysis results which were inconsistent with encephalitis. WBC, white blood cell; RBC, red blood cells; CSF, cerebrospinal fluid; HSV, herpes simplex virus; PCR, polymerase chain reaction

CSF test	Result	Reference range
Glucose	93	40-75 mg/dL
Protein	47	14-45 mg/dL
WBC	0	
RBCs	0.059	
%crenated RBCs	10	
%RBCs	90	
%neutrophils	0	
%lymphocytes	0	
%monocytes	0	
%eosinophils	0	
%blasts	0	
%other cell lines	0	
CSF culture	Negative	
CSF Gram stain	Negative	
CSF HSV PCR	Negative	

**Figure 4:**
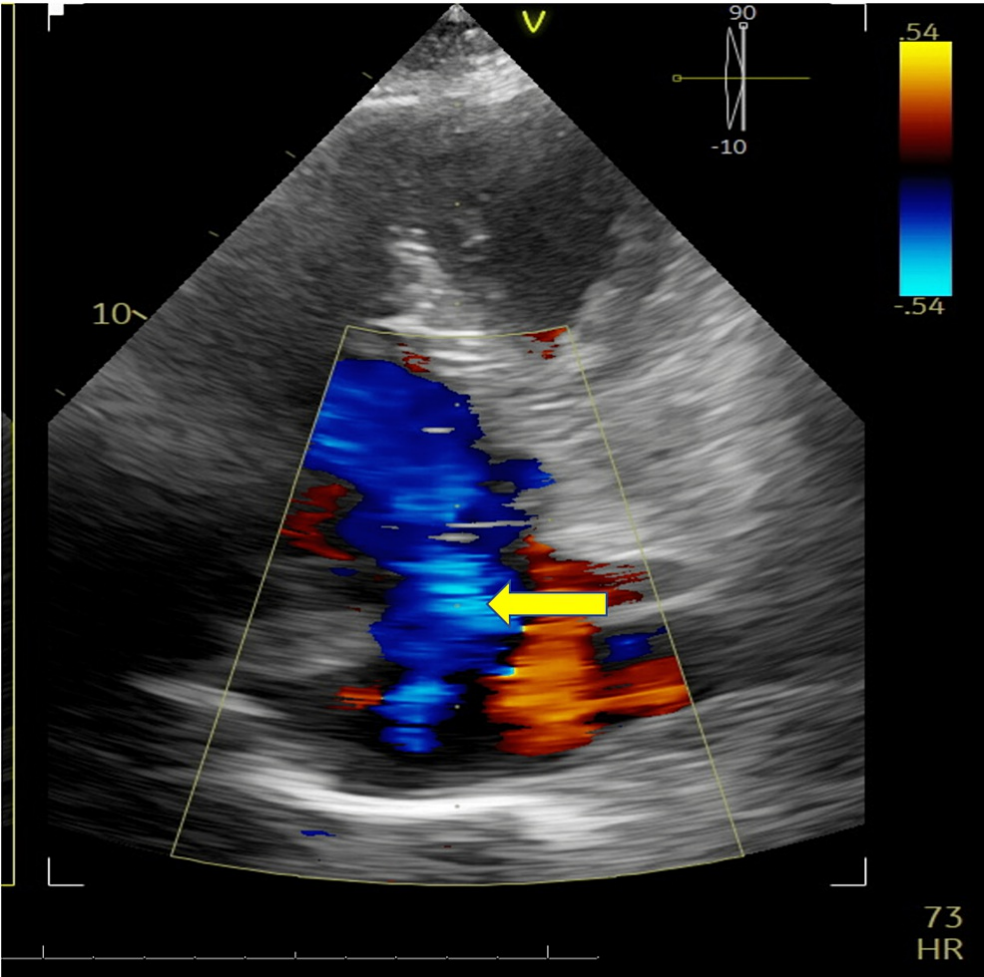
TTE with color Doppler demonstrating severe tricuspid regurgitation. The yellow arrow highlights regurgitant flow of blood from the right ventricle into the right atrium during systole. TTE, transthoracic echocardiogram

Given the CSF findings, acyclovir was discontinued. As part of the work-up for stroke, an echocardiogram was obtained (see Figure 4). The echocardiogram showed moderate to severe tricuspid valve regurgitation. In addition, it raised concern for a tricuspid mass vs. vegetation. 

**Figure 5:**
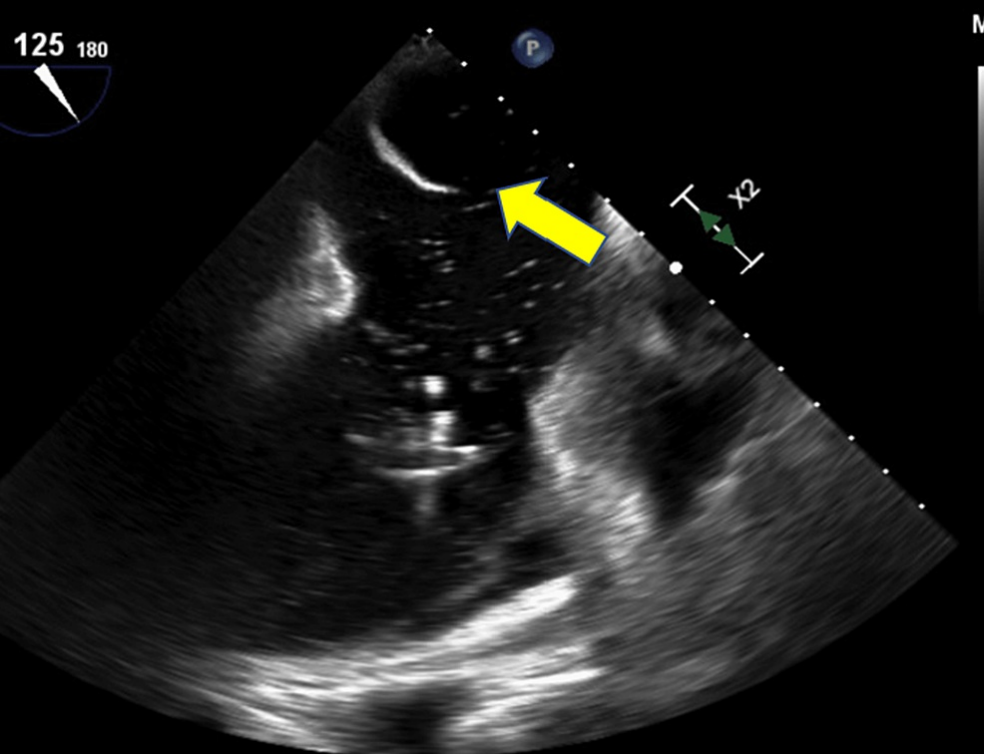
TEE showing a possible atrial septal defect. The yellow arrow indicates what appears to be a communication between the left atrium and the right atrium concerning for an atrial septal defect. TEE, transesophageal echocardiogram

A transesophageal echocardiogram (TEE) redemonstrated severe tricuspid valve regurgitation. Saline contrast injection and color Doppler evaluation were suggestive of a large atrial septal defect (ASD) (Figure 5).

**Figure 6:**
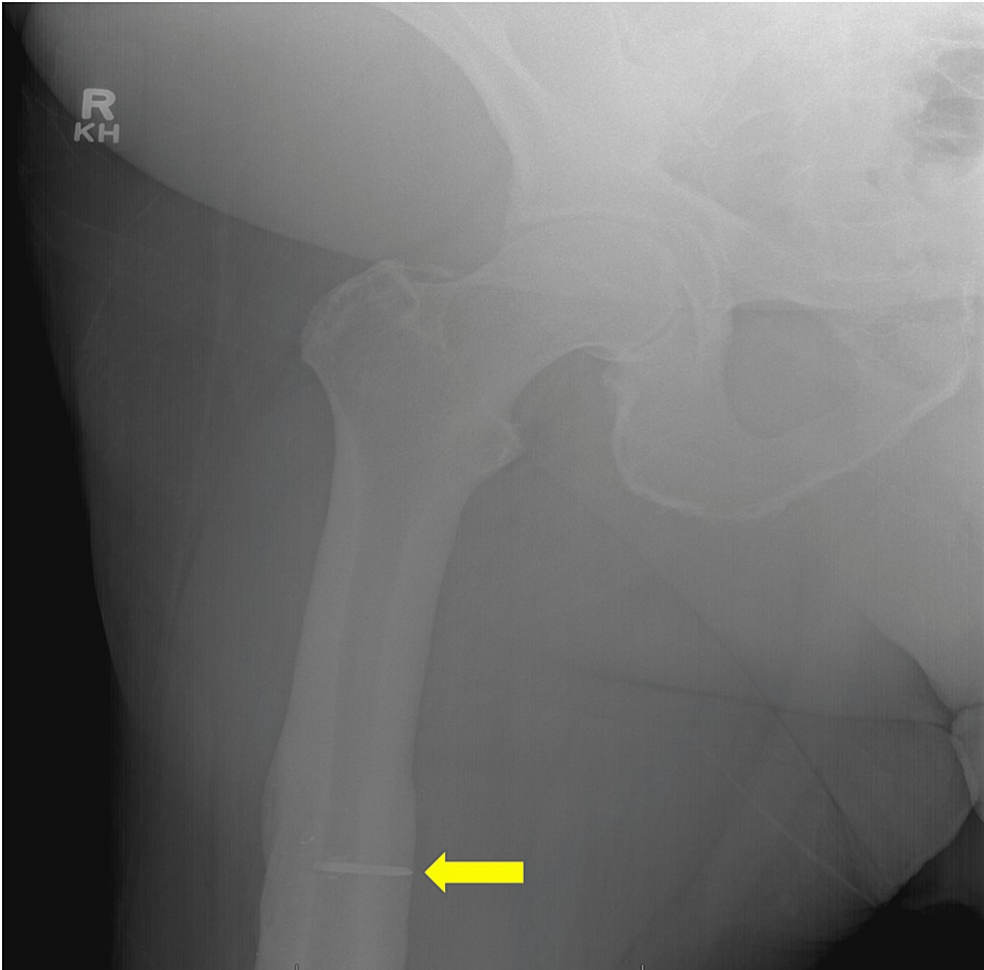
Right femur X-ray showing retained screw fragment in right mid femoral diaphysis from prior ORIF. The yellow arrow indicates the retained screw fragment in the right femoral diaphysis. ORIF, open reduction and internal fixation

Therefore, there was suspicion that the tricuspid “mass” was a dislodged atrial septal closure device. Due to concern for thromboembolism as a cause of his encephalopathy, the patient was thus placed on an IV heparin infusion. The patient had a drop in his platelet count while on IV heparin, and although he had baseline thrombocytopenia, the patient was placed on bivalirudin due to concern for heparin-induced thrombocytopenia (4T score was 4). A serotonin release assay was sent. Of note, before undergoing MRI, the patient underwent X-rays of the abdomen, bilateral humerus, and femur to check for foreign bodies. The right femoral X-ray (Figure 6) showed findings consistent with a prior right femoral ORIF (open reduction and internal fixation) with retained screw fragments. It was, therefore, conjectured that the mass adherent to the tricuspid valve could be an IVC filter placed in the setting of pulmonary embolism before an orthopedic procedure.

**Figure 7:**
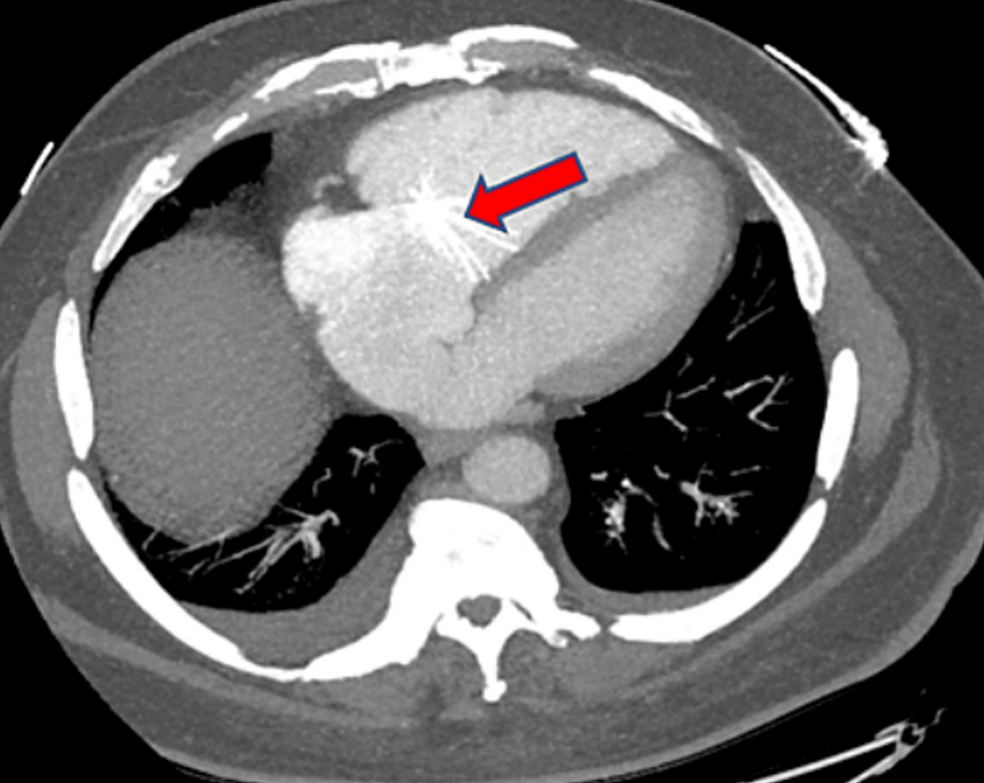
Cardiac CT demonstrating migrated IVC filter in the right ventricle. Red arrows highlight the migrated IVC filter at the tricuspid valve. IVC, inferior vena cava

The valve was visualized under fluoroscopy and the appearance of the tricuspid mass was found to be more consistent with an IVC filter. A cardiac CT scan (Figure 7) was obtained that showed findings that were discrepant with the report of an ASD on the TEE. Due to concern for heparin-induced thrombocytopenia, there were questions about the feasibility of cardiac surgery as this diagnosis would be a contraindication for heparin use during cardiopulmonary bypass. Fortunately, the serotonin release assay was ultimately negative.

A decision was made to proceed with cardiac surgery for IVC filter removal, possible tricuspid valve repair, and ASD closure. On day 21 of his admission, the patient underwent IVC filter removal, tricuspid valve repair with the attachment of torn posterior and anterior leaflets to the tricuspid annulus, closure of commissure between the posterior and anterior tricuspid leaflets, and placement of a #30 Edwards Tricuspid annuloplasty band. No ASD was found. Instead, the patient had a prominent eustachian valve. Over the subsequent weeks, the patient had marked clinical improvement in his right heart failure as well as his mentation and speech.

## Discussion

IVC filters can migrate to the right heart and beyond resulting in damage to structures in and around the heart. In addition to tricuspid valve migration [6], other cardiovascular complications of IVC filter migration include ventricular arrhythmias [7] and embolization to the pulmonary artery [8]. Although there are other case reports of IVC filter migration to the tricuspid valve, to our knowledge, this is the first case treated with tricuspid valve annuloplasty. This is one of the few cases of IVC filter migration presenting with paradoxical embolism. In addition, other facts about this case are worth highlighting.

The patient’s presentation with acute encephalopathy, MRI findings suggestive of stroke as well as right to left shunting of bubble contrast on TEE made a compelling case for paradoxical emboli through an ASD. On the transesophageal echocardiogram, there was movement of bubble contrast from the right atrium to the left atrium. On Doppler echocardiography, there was movement of blood across the inter-atrial septum. Furthermore, there was what appeared to be a communication between the right atrium and the left atrium. Strangely, the CT heart did not redemonstrate the ASD. In addition, an ASD was not identified during the surgical removal of the IVC filter and tricuspid valve repair. The appearance of bubble contrast in the left atrium could not be explained by an atrial septal defect as evidenced by findings during cardiac surgery.

A possible explanation is extracardiac shunting of bubble through for example an arteriovenous malformation. Classically, an intracardiac shunt is diagnosed when bubbles appear in the left atrium within 3-5 cardiac cycles [9]. The appearance of bubbles in the left heart later in the (>5 beats) after right chamber opacification is typically indicative of an extracardiac shunt. However, bubbles can traverse a sizeable pulmonary shunt within 3-5 cardiac cycles resulting in a false positive study for an intracardiac shunt [9]. In theory, the high output state in association with, for example, an arteriovenous malformation, would allow for quicker bubble passage into the left heart. An arteriovenous malformation is also a potential conduit for paradoxical emboli, especially in a patient without evidence of an intracardiac shunt in the operating room.

## Conclusions

In conclusion, migration to the right heart is a potential complication of IVC filters that can present with tricuspid regurgitation and right-sided heart failure. The filter can sometimes mimic a tricuspid valve vegetation or a mass. Echocardiography can be falsely positive for atrial septal defects. When CT imaging or direct visualization disagrees with the echocardiographic finding of an ASD, other causes of right-to-left bubble passage should be considered. Extracardiac shunting should be considered in patients in whom there is a concern for paradoxical emboli when an intracardiac shunt has been ruled out.
